# Systems biology approach uncovers candidates for kidney-heart interorgan crosstalk after myocardial infarction

**DOI:** 10.1038/s41598-025-30712-z

**Published:** 2025-12-05

**Authors:** Hanna Wolf, Svenja Kupsch, Virginia K. Haacke, Lucas Bacmeister, Susanne Weber, Ingo Hilgendorf, Till Keller, Ralf Dechend, Tobias B. Huber, Dirk Westermann, Diana Lindner

**Affiliations:** 1 Department of Cardiology and Angiology, University Heart Center Freiburg-Bad Krozingen - University of Freiburg, Faculty of Medicine, University of Freiburg, Freiburg, Germany; 2 Institute of Medical Biometry and Statistics, Medical Center - University of Freiburg, Faculty of Medicine, University of Freiburg, Freiburg, Germany; 3 Experimental and Clinical Research Center, A cooperation between the Max‐Delbrück‐Center for Molecular Medicine in the Helmholtz Association, Charité ‐ Universitätsmedizin Berlin, HELIOS Clinic Berlin-Buch, Berlin, Germany; 4 III. Department of Medicine, University Medical Center Hamburg-Eppendorf, Hamburg, Germany; 5 Hamburg Center for Kidney Health (HCKH), University Medical Center Hamburg-Eppendorf, Hamburg, Germany

**Keywords:** Ischemia, Heart-kidney crosstalk, Myocardial infarction, Systems biology, GDF-15, Cardiorenal syndrome, Cardiology, Computational biology and bioinformatics, Diseases, Nephrology

## Abstract

Myocardial infarction (MI) and subsequent heart failure are frequently accompanied by chronic kidney disease, further impairing outcomes and complicating treatment. To better understand heart-kidney crosstalk, we used RNA sequencing data to infer interorgan signalling after experimentally induced MI in mice, focusing on secreted biomolecules and interorgan cross talk that may drive cardiorenal syndrome (CRS). To assess acute and chronic effects, we examined kidneys at 5d and 28d post-MI, evaluating changes in renal gene expression and fibrosis. During the acute phase 5d post-MI, several genes inferred to target kidney receptors were highly upregulated in the cardiac infarct zone, with *Postn* and *Spp1* being the most probable ligands. However, only minor changes in gene expression were detected in the kidney 5d post-MI. At 28d post-MI, renal fibrosis and the number of differentially expressed genes (DEGs) in the kidney increased. Gene ontology enrichment suggested metabolic adaptions as part of a long-term response. Based on upregulated DEGs in kidney 28d post-MI, we suggest two kidney-to-heart interactions: *Slitrk6*-*Ptprs* and *Gdf15*-*Tgfbr2. *In vitro, GDF-15 treatment of human cardiac fibroblasts induced pro-fibrotic gene expression, mirroring in vivo changes in the heart. Our data suggest that MI in mice elicits minimal acute response in kidney but triggers chronic transcriptional and pro-fibrotic changes in kidney, potentially driven by altered renal metabolism. The inference of interorgan signalling molecules such as GDF-15 points towards candidate mediators of CRS and provides a basis for future mechanistic and clinical studies.

**Supplementary Information:**

The online version contains supplementary material available at 10.1038/s41598-025-30712-z.

## Introduction

Acute myocardial infarction (MI) and subsequent heart failure (HF) are frequently accompanied by chronic kidney disease (CKD), a condition that worsens patient prognosis and complicates treatment. The analysis of the interplay between heart and kidney following MI could reveal pathways that contribute to the mutual progression of both cardiac and renal damage, presenting new molecular options for future therapeutic interventions.

The pathophysiological relationship between heart and kidneys is conceptualized as cardiorenal syndrome (CRS) and classified into five subtypes. Of those, CRS types 1 and 2 represent scenarios primarily driven by acute and chronic HF, respectively.^1^ While a natural decline in kidney function is a normal part of aging, following MI, kidney function declines at an accelerated rate. A first ischemic cardiac event accelerates the natural deterioration of kidney function, with post-MI patients experiencing a 2.5-fold faster deterioration if multiple cardiovascular risk factors are present.^2,3^ CRS type 1 can develop after acute MI driven by multiple mechanisms. These include: (1) cardiomyocyte necrosis accompanied by the release of damage-associated molecular patterns (DAMPs), (2) hemodynamic alterations leading to acute HF, (3) subsequent neurohumoral activation, and (4) secretion of factors from injured cardiac tissue. In CRS type 2, derived from chronic HF, cardiac injury often results in a progressive decline in renal function and increased mortality. This progression is mediated not only by reduced hemodynamic alterations, but also by secreted factors.^1,4^ It is known that cardiac-secreted factors, such as Atrial and B-Type Natriuretic Peptides, can modulate peripheral organs, including the kidneys.^5,6^ Additionally, cytokines actively released from the diseased heart may not only exacerbate cardiomyocyte damage but also contribute to systemic organ damage.^7^ However, the exact pathways through which these actively heart-released molecules mediate renal damage remain poorly understood.

Given the profound impact of cardiac injury on renal function, it is equally important to recognize that renal dysfunction can also exacerbate cardiovascular diseases. In particular, renal dysfunction is known to increase the risk of cardiovascular diseases.^8,9^ Patients with CKD often exhibit pathological changes in the myocardium, such as myocardial fibrosis and cardiac hypertrophy, hallmarks of uremic cardiomyopathy.^10^ Left ventricular hypertrophy is present in about one-third of all CKD patients, and renal disease is considered a major risk factor for cardiovascular complications after MI.^11^ These findings underscore the complex and bidirectional nature of the relationship between kidney and heart.

Here we aim to explore the molecular crosstalk between heart and kidneys after MI to propose pathways which potentially drive disease progression. Therefore, we conducted an exploratory, bioinformatic-based analysis using RNA sequencing data to study heart-kidney interorgan crosstalk after MI in mice. We aim to infer specific ligand-receptor interactions from the injured heart to the kidney or from a damaged kidney back to the heart. Those molecular targets that may drive the disease progression of the CRS may represent new therapeutic targets to mitigate both cardiac and renal damage, ultimately improving patient outcomes.

## Material and methods

### Animal model

To experimentally induce MI, male C57BL/6 J (B6) wild-type mice aged 8–11 weeks and weighing 22–28 g underwent permanent ligation of the left anterior descending coronary artery (LAD) as described previously.^12,13^ Mice were anesthetized by inhalation of isoflurane (2–3%) after receiving a subcutaneous injection of buprenorphine (0.05 mg/kg). Sham-operated animals underwent the same procedure without coronary ligation. Following surgery, mice recovered for either 5 or 28d before hemodynamic measurements were performed, with data previously published.^12^ Mice were scarified at the end of hemodynamic measurement using cervical dislocation, and organs were explanted. Left ventricular (LV) tissue was collected, and in MI mice, further dissected to isolate the infarct zone (IZ) and the unaffected remote zone (RZ). Kidneys and LV tissue samples were immediately snap-frozen in liquid nitrogen and stored at −80 °C for further analysis. All animals were bred in the institutional research animal facility of the University Clinics Hamburg Eppendorf. Experiments were approved by the Hamburg Ministry of Health and Consumer protection, Hamburg, Germany (G15/060) and all methods were performed as approved and in accordance to the *Guide for the Care and Use of Laboratory Animals* and to the ARRIVE guidelines.

### Human cardiac fibroblasts

Human cardiac fibroblasts were obtained by outgrowth from endomyocardial biopsies as described previously.^14,15^ Cells were cultured in Iscove’s basal medium (Biochrom AG) containing 10% human serum, 10% fetal calf serum (Biochrom AG), 100 U/mL penicillin, and 100 µg/mL streptomycin (Sigma-Aldrich) and grown in 6-well plates until confluence. The stimulation experiments with those primary cells were carried out between passages 2 and 3 in 24 well plates.^15,16^ Before stimulation, cells were serum-starved in serum-reduced medium, Iscove’s basal medium with 0.5% fetal calf serum, 100 U/mL penicillin, and 100 µg/mL streptomycin overnight. For stimulation, cells were treated with 100 ng/ml GDF-15 (Peprotech) in serum-reduced starving medium for 24 h. Controls were treated equally without the addition of GDF-15. Finally, cells were lysed in RLT buffer (Qiagen) containing 1% beta-mercaptoethanol and stored at −80 °C for subsequent RNA isolation.

### RNA Isolation

To isolate total RNA from snap-frozen tissue, it was first disrupted in QIAzol by stainless steel beads utilizing a tissue lyser II (Qiagen) followed by chlorophorm extraction. RNA containing aqueous phase was mixed with ethanol and further purified using the miRNeasy mini kit (Qiagen) according to the manufacturer’s protocol. To obtain total RNA from cells, the RNeasy Mini Kit (Qiagen) was used according to the manufacturer’s protocol. For both, tissue and cell culture samples, genomic DNA contamination within the isolated RNA was avoided applying DNase-I (Qiagen) directly on the column during the purification protocol. RNA concentration was determined using a Nanodrop 2000c spectrophotometer (Thermo Fisher Scientific). RNA was stored at −80 °C.

### Gene expression analysis

RNA was reversely transcribed into cDNA using the high-capacity cDNA kit (Life Technologies). For tissue samples, 1 µg of RNA was used, for cells 0.25 µg was used for cDNA synthesis. Reverse transcription was carried out at 37 °C for 2 h followed by an inactivation step of 5 min at 85 °C. The cDNA was diluted with water before gene expression analysis (10 ng/µL for tissue samples, 1.25 ng/µL for cell culture samples).

To examine gene expression of target genes, quantitative real-time PCR was performed using 2.5 µL TaqMan gene expression master mix (Thermo Fisher Scientific) and 0.25 µL TaqMan gene expression assay (Supplementary Table S1). A volume of 1 µL cDNA was used as template in a final volume of 5 µL. Each sample was analysed in duplicates. Real-time PCR was performed on a QuantStudio™ 7flex or QuantStudio™ 7pro system (Thermo Fisher Scientific) using the QuantStudio™ software v1.3 or Design and Analysis software 2.6.0, respectively. Gene expression of *Cdkn1b* or/and *18S* was used as endogenous control to calculate ∆Ct values of target genes. To determine absolute gene expression, the formula 2^−∆Ct^ was used and obtained values were plotted as x-fold to *Cdkn1b*, *18S,* or the mean of both. To determine relative gene expression, mean of ΔCt values of the respective control group was used to calculate normalized ΔΔCt values. Relative gene expression data were determined using the formula 2^−ΔΔCt^ and plotted as x-fold to the respective control.^17^.

### MACE RNA sequencing analysis

#### 3’mRNA sequencing

Massive Analysis of cDNA Ends (MACE) is a 3’mRNA sequencing method based on the analysis of Illumina reads derived from fragments that originate from 3’ mRNA ends.^18^ RNA samples were processed by GenXPro GmbH (Frankfurt) using the Rapid MACE-Seq kit according to the manual of the manufacturer. Fragmented RNA underwent reverse transcription using barcoded oligo(dT) primers containing TrueQuant unique molecular identifiers, followed by template switching. PCR amplified libraries were purified by solid phase reversible immobilization beads and subsequent sequencing was performed using the Illumina platform NextSeq 500. Unprocessed sequencing reads were adapter-trimmed and quality-trimmed using Cutadapt (v4.6). The reads were mapped to the mouse genome (mm10) and transcripts were quantified by HTSeq (Supplementary Table S2). Differential gene expression was determined using DESeq2 (v1.38)^19^ and plotted as Volcano plots using Graph Pad Prism (GraphPad Software, USA). Principal component analysis (PCA) was performed on variance-stabilized DESeq2 data and visualized through MultiQC (v1.16) (Supplementary Figure 1A).

#### Gene ontology (GO) enrichment analysis

GO enrichment analysis was performed using the R plugin *clusterProfiler* (v4.12.6) and its function *enrichGO* with ENSEMBL identifiers and the organism database *org.Mm.eg.db* (v3.19.1). Enrichment of GO terms was tested using a hypergeometric over-representation test, which compares the observed frequency of genes associated with each GO term to the expected frequency under a random distribution. For the analysis, all differentially expressed genes (DEGs) with an adjusted *p*-value < 0.05 were used and tested against all detected genes in the dataset. Resulting *p*-values were adjusted with Benjamini–Hochberg correction. The resulting significantly enriched GO terms were refined using REVIGO^20^ (v1.8.1) with default setting. GO terms were plotted as dotplot using the R plugin *ggplot2* (v3.5.1).

#### Inference of ligand-receptor interactions

Potential ligand-receptor interactions between kidney and heart were inferred using the R plugin *CellChat* (v2.1.2). For this purpose, a transcript per million (tpm) count matrix for all kidney and heart samples was used and the sources of samples were referred as cell types (Kidney sham, Kidney 5d post-MI, Heart sham, Heart IZ 5d post-MI). The mouse-specific *CellChat* database (CellChatDB.mouse) was subset, and overexpressed genes and interactions were identified. To identify potential interactions, data was projected onto the mouse protein–protein interaction (PPI) network. Communication probabilities were computed exclusively based on “secreted signalling” using the *triMean* method. Specific interactions were plotted as bubble plot using *netVisual_bubble*. To plot significantly upregulated ligands in the kidney 28d post-MI, the *CellChat* database was used. All ligands were compared with the DEGs in the kidney 28d post-MI. The tpm count of each animal was normalized to the mean of the sham group and plotted as a heatmap using the R plugin *Complex-Heatmap* (v2.22.0).

#### Analysis of ligand and receptor expression in public single-cell datasets

To evaluate the cell-type-specific expression of inferred ligands and receptors, we analysed publicly available human single-cell RNA sequencing datasets for the kidney and heart. For the kidney, we used data from healthy kidneys published by Stewart et al.^21^, and for the heart, data from infarcted and healthy hearts published by Kuppe et al.^22^. Cell type–annotated single-cell RNA sequencing data were obtained as processed files from the respective authors’ public repositories. For each gene, normalized expression values were averaged across annotated cell types, and expression of inferred ligands and receptors was visualized as heat map using *Complex*-*Heatmap* (v.2.22.0) package in R.

### Histological analyses

Snap-frozen kidney samples were sectioned at 10 µm thickness using a cryostat. Fibrosis was visualized by Picrosirius Red staining with the Picrosirius Red Stain Kit (ScyTek Laboratories). Briefly, sections were hydrated using a descending ethanol series, counterstained for 90 s in 0.5 × Mayer’s hemalum solution (Merck Millipore) diluted in distilled water and then blued in warm tap water for 10 min. Collagen staining was performed as described in the manufacturer’s protocol. Finally, sections were dehydrated in an ascending series of ethanol followed by incubation in NeoClear (Sigma). Slides were mounted in Entellan (Sigma). Images were captured using a BZ 9000 microscope (Keyence) with 20 × CFI PL APO Lbd (NA = 0.75) or 60 × CFI PL APO Lbd H (NA = 1.4) objective using the BZ II Analyzer software. Fibrosis within renal tissue was additionally imaged in the red channel using a Texas Red filter and quantified in the fluorescent images using FIJI (v2.14.0). Fibrotic area was assessed in two non-consecutive kidney sections each quantified by 3–6 randomly selected images taken in the region of the glomeruli in the renal cortex. The fibrotic area in each section was calculated as the ratio of the fibrotic tissue area to the total tissue area. The mean value per animal was then calculated and used for statistical analysis, which is presented in the graph with mean ± SD.

### Statistics

Statistical analysis of gene expression data between two groups was performed using an unpaired two-tailed t-test, assuming normal distribution of data. For comparisons involving more than two groups, Bonferroni correction was applied to adjust p-values for multiple comparisons. Data are presented as 2^−ΔΔCt^ with geometric mean ± 95% confidence interval (CI). The analysis of RNA sequencing data is described in the respective section.

## Results

### Transcriptomic changes in the heart and kidney 5d post-MI

**Fig. 1:**
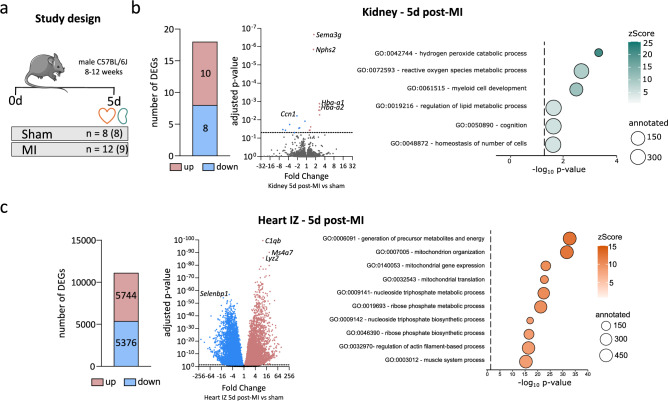
Transcriptomic changes in the heart and kidney 5d post-MI. (**a**) Permanent LAD ligation was performed in 12 mice, while 8 mice underwent sham surgery. Tissues from heart and kidney were collected 5d after surgery. RNA sequencing was performed on RNA from cardiac infarct zones (IZ) and kidneys from 9 randomly selected mice at 5d post-MI, as well as on RNA from hearts and kidneys from all 8 sham-operated mice. Differentially expressed genes (DEGs) were identified using DESeq2 with the Wald test and Benjamini–Hochberg correction (adjusted* p*-value < 0.05). (**b**) In the kidney, 18 DEGs were detected and visualized in a volcano plot. The x-axis represents fold change, and the y-axis represents the adjusted *p*-value. Up- and downregulated DEGs are shown in red and blue, respectively. All significantly enriched GO terms were refined using REVIGO to reduce redundancy. Dot size represents the number of annotated genes, and colour indicates the zScore, reflecting GO term enrichment. (**c**) More than 11,000 DEGs were identified in the IZ of the heart and displayed in a volcano plot. The x-axis represents fold change, and the y-axis represents the adjusted *p*-value. GO term analysis was performed on the DEGs and refined using REVIGO. Dot size represents the number of annotated genes, and colour indicates the zScore of enrichment.

To identify DEGs in the heart and kidney 5 days after induced MI, we performed permanent ligation of the LAD in 12 male B6 wild-type mice. Eight additional mice underwent sham-surgery without LAD ligation and served as controls. RNA sequencing was conducted on cardiac IZ and the corresponding kidneys from nine randomly selected mice at 5d post-MI, as well as on LV tissue and kidneys from eight sham-operated mice (Fig. 1A).

In the kidney, 10 genes were significantly up- and 8 genes were significantly downregulated 5d post-MI (Fig. 1B, Supplementary Table S3). Five out of the 10 upregulated DEGs in the kidney 5d post-MI were haemoglobin-related (*Hba-ps3*, *Hbb-bt*, *Hba-a2*, *Hba-a1*, *Hbb-bs*). Despite the low number of DEGs, the PCA revealed a clear separation of the 5-day post-MI kidney samples from the sham controls (Supplementary Fig. 1A). To further validate the RNA sequencing results, upregulation of *Nphs2* and *Hba-a1* was confirmed by gene expression analysis (Supplementary Fig. 1B). To categorize the identified DEGs into functional groups, GO enrichment analysis was conducted on the kidney RNA-seq dataset using DEGs as input. To minimize redundancy, significantly enriched GO terms were refined using the REVIGO tool. Given the limited number of DEGs, only 2–4 of them contributed to each enriched GO term. Among these, the GO term "hydrogen peroxide catabolic process" displayed the highest zScore, primarily driven by the DEGs *Hba-a1*, *Hbb-b1*, *Hbb-bt*, and *Ccn1* suggesting processes related to oxidative stress.

In the heart, the IZ exhibited 5744 significantly up- and 5376 downregulated genes 5d post-MI (Fig. 1C, Supplementary Table S4). The subsequent GO term analysis of these DEGs revealed a strong enrichment of processes related to mitochondrial function and energy metabolism. REVIGO was used to refine the analysis removing redundant GO terms. Among the 10 most significantly enriched GO terms were “generation of precursor metabolites and energy”, “mitochondrion organization”, “mitochondrial gene expression”, and “mitochondrial translation”. Additionally, terms involved in nucleoside triphosphate and ribose phosphate metabolism were significantly enriched. These findings suggest an active metabolic and mitochondrial response to ischemia in the IZ during post-MI remodelling.

Taken together, compared to the heart, gene expression in the kidney showed only minor changes during the acute phase after MI. However, the kidney exhibited upregulation of haemoglobin-related genes and GO term enrichment in processes related to oxidative stress, while the heart exhibited a strong upregulation of mitochondrial and energy metabolism-related genes as response to ischemia at 5d post-MI.

### Inference of heart-kidney interorgan crosstalk 5d post-MI

**Fig. 2:**
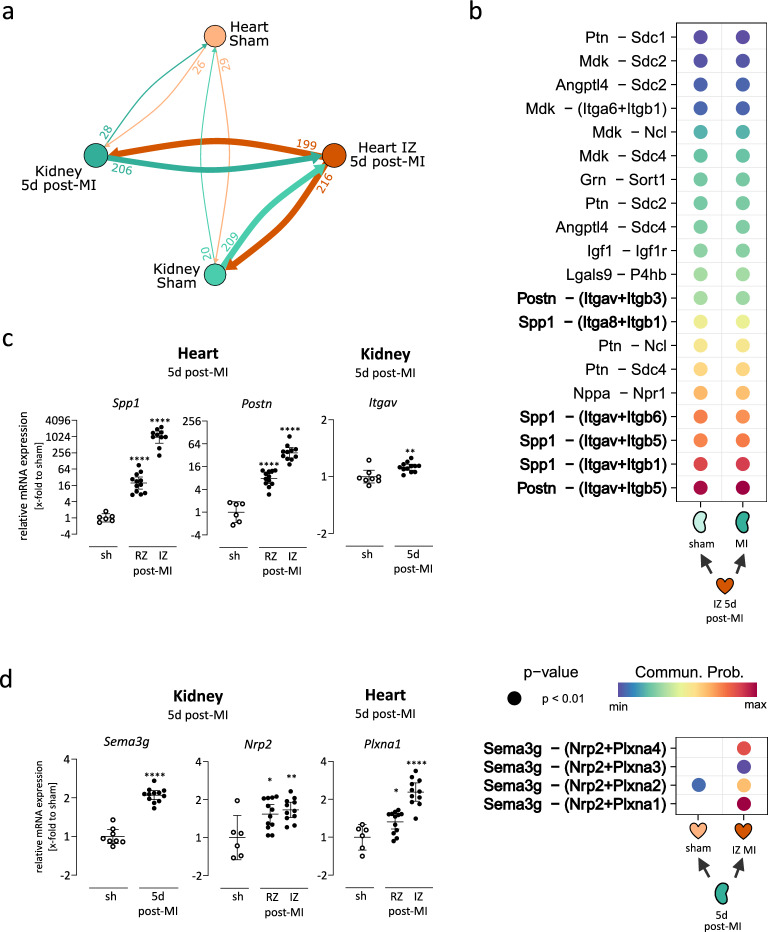
Inferred interorgan signalling between heart and kidney 5d post-MI. (**a**) Interorgan communication between the heart and kidney post-MI was analysed using the R plugin *CellChat*, restricted to secreted signalling interactions. Crosstalk was primarily driven by extensively altered gene expression in the infarct zone (IZ) 5d post-MI. (**b**) Inferred interactions were restricted to those with significantly upregulated ligands. The bubble plots display the 20 most probable inferred interactions in the heart (top) and the inferred interactions in the kidney (bottom). In the kidney, only one significantly upregulated ligand was detected. (**c**) Gene expression of *Postn* and *Spp1* was measured in the remote zone (RZ) and IZ, while their inferred receptor *Itgav* was assessed in the kidney 5d post-MI. Gene expression was normalized to *Cdkn1b* and sham controls. (**d**) Gene expression of *Sema3g* was measured in the kidney, while the gene expression of its inferred receptors *Nrp2* and *Plxna1* was assessed in the RZ and IZ 5d post-MI. Gene expression was normalized to *Cdkn1b* and sham controls. Gene expression data are plotted as 2^-ΔΔCt^ (geometric mean ± 95% CI, unpaired two-tailed *t*-test with Bonferroni correction. *, **, ****; *P* < 0.05, 0.01, 0.0001).

Next, we aimed to investigate potential interactions between infarcted heart and kidneys. Therefore, we analysed interorgan signalling using the *CellChat* R package based on the RNA sequencing data as input. In Fig. 2A, the number of proposed interactions is plotted and visualized by thickness of connecting lines. Interactions involving heart-derived secreted factors targeting receptors in the kidney are shown in orange, while kidney-derived secreted signals addressing receptors expressed in the heart are shown in green (Supplementary Table S5). Compared to healthy LV tissue, the IZ 5d post-MI expressed more than a sixfold higher number of secreted factors inferred to be ligands for receptors expressed in the kidney (199 and 216 from IZ vs. 26 and 29 from sham heart). Similarly, interactions based on kidney-derived ligands to the IZ 5d post-MI (209 from sham kidney vs. 206 from 5d post-MI kidney) were also more frequent than to sham LV tissue (20 from sham kidney vs. 28 from 5d post-MI kidney). The number of proposed interactions is primarily driven by upregulated ligands, but also receptors, in the injured cardiac tissue, while both heart and kidney act as senders as well as receivers.

We aimed to focus on the most relevant post-MI interactions in the interorgan crosstalk from injured heart to kidney. Therefore, the ligands from the cardiac IZ 5d post-MI for all inferred interactions were restricted to upregulated DEGs between sham and IZ. This yielded 131 proposed interactions, of which the 20 most probable ones are displayed as a bubble plot in Fig. 2B. The most probable inferred interactions were driven by the cardiac ligands periostin (*Postn*) and osteopontin (*Spp1*). In line with the RNA sequencing data, the subsequent gene expression analysis of these genes validated a significant increase in their expression 5d post-MI in both the RZ and IZ. Furthermore, the targeted receptor *Itgav* was slightly but significantly upregulated in the kidney 5d post-MI in the gene expression analysis (Fig. 2C). In the RNA sequencing analysis, *Itgav* was not significantly upregulated, although it displayed a non-adjusted p-value of 0.016 (Supplementary Table S3). However, the upregulation observed in both analyses was low. Furthermore, multiple inflammation-related pathways were inferred, including CCL and CXCL chemokine signalling, the interaction of IL6–IL6R and IL1–IL1R, as well as the activation of protease-activated receptors (Supplementary Table S5).

Next, we analysed the effect of the affected kidney back to the heart. Therefore, we additionally analysed kidney-derived secreted factors 5d post-MI and identified *Sema3g* as the only significantly upregulated ligand (Fig. 2C). In the RNA sequencing data, all receptors targeted by *Sema3g* in the heart, except *Plxna2*, were upregulated in IZ compared to healthy LV tissue (Supplementary Table S4). Upregulation of *Sema3g* in the kidney 5d post-MI, as well as *Nrp2* and *Plxna1* in the infarcted heart 5d post-MI, was confirmed by gene expression analysis (Fig. 2D).

To investigate the cell-type specificity of the inferred ligand–receptor interactions, we analysed their expression in publicly available human single-cell datasets^21,22^. In the healthy kidney, the ligand *Sema3g* showed highest expression in *descending vasa recta endothelial cells* located in the renal medulla, whereas the corresponding inferred receptors were distributed across several cardiac cell types (Supplementary Fig. 2A). For the heart, we examined a dataset including control and myocardial infarction samples (Kuppe et al.), where the expression of *Postn* and *Spp1* was markedly increased within the infarct zone (Supplementary Fig. 2B). *Postn* expression was broadly elevated across nearly all cardiac cell types, while *Spp1* upregulation was most prominent in myeloid cells. In addition, the ligand *Angptl4* was strongly upregulated in multiple cardiac cell populations.

Due to the low number of DEGs in kidney 5d post-MI, we suggest that kidney dysfunction as a result of MI may develop later than 5d post-MI. This implicates that DAMPs from the injured heart have no major effects on the kidneys since their effects should have been detectable in RNA-sequencing 5d post-MI. However, actively secreted mediators, such as *Postn* and *Spp1* released by the IZ at 5d post-MI, might influence the kidney and drive the progression of kidney dysfunction.

### Long-term consequences of MI on kidney

**Fig. 3:**
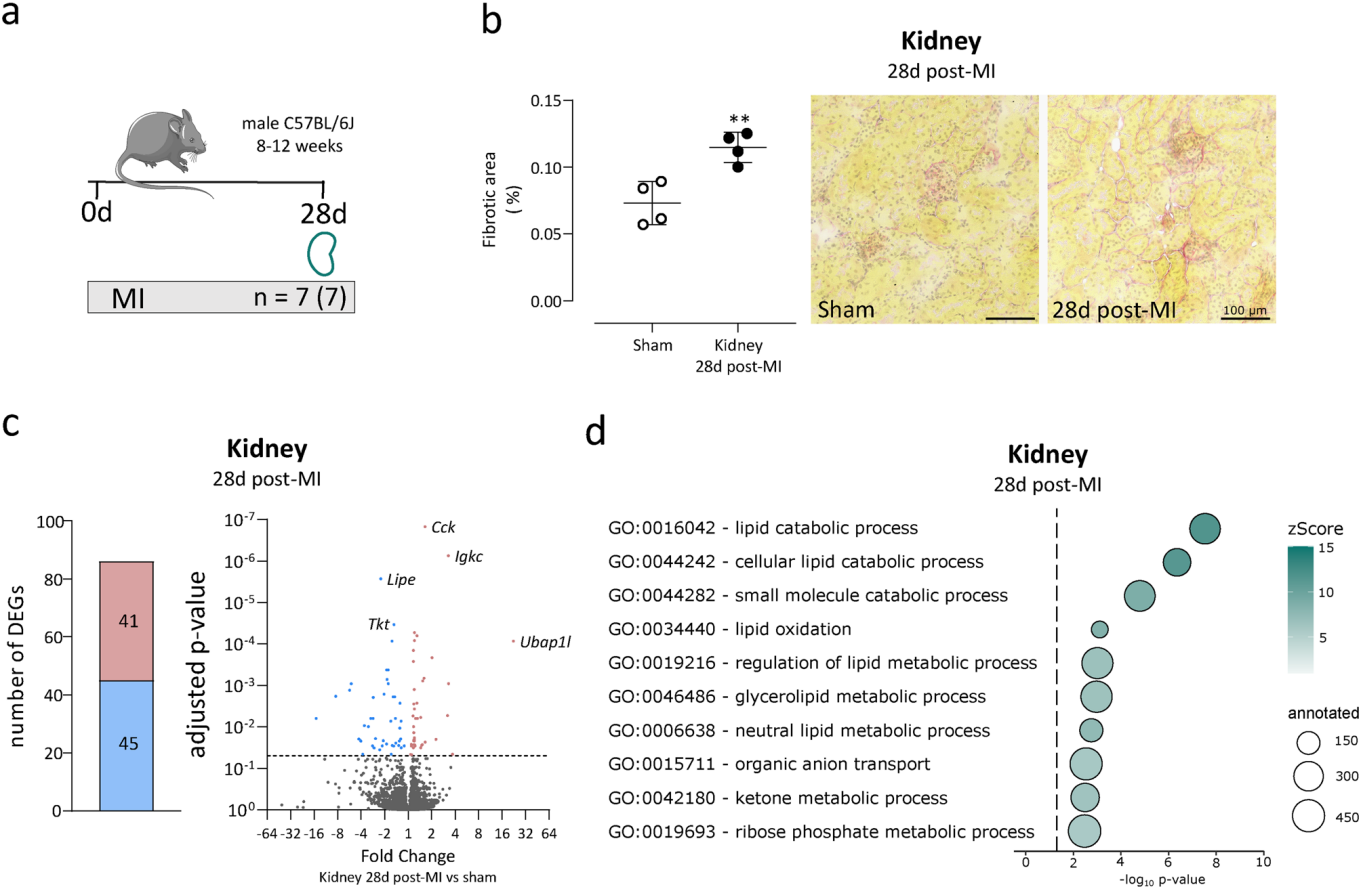
Kidney fibrosis and transcriptomic changes 28d post-MI. (**a**) Permanent LAD ligation was performed in 7 mice, and tissues were collected 28d post-MI. RNA sequencing was conducted on kidney RNA from these mice (n = 7), while kidneys from sham-operated mice at the 5d post-MI time point served as controls. (**b**) Fibrosis was quantified in Picrosirius Red-stained cryosections from kidneys of sham-operated mice (n = 4) and mice 28d post-MI (n = 4). The fibrotic area was significantly increased in kidneys 28d post-MI (mean ± SD, unpaired two-tailed t-test). (**c**) Transcriptome analysis was performed via RNA sequencing of kidney tissue at 28d post-MI. DEGs were identified using DESeq2 with Wald test and Benjamini–Hochberg correction (adjusted *p*-value < 0.05). A total of 86 DEGs were detected. The Volcano plot displays fold change (x-axis) and adjusted p-value (y-axis). Upregulated DEGs are marked in red, downregulated in blue. (**d**) GO term analysis was conducted using the identified 86 DEGs in kidney 28d post-MI as input. All significantly enriched GO terms with more than 5 annotated DEGs were refined using REVIGO. The 10 most significantly enriched terms are shown as dot plot, in which dot size represents the number of annotated genes, and colour indicates the zScore, reflecting GO term enrichment.

So far, we have found that acute MI hardly alter gene expression in the kidney. However, several secreted factors from the IZ were inferred to target receptors in the kidney, potentially contributing to long-term effects on the kidneys. To investigate chronic effects after MI, we next analysed kidneys at 28d post-MI as depicted in Fig. 3A.

As both *Postn* and *Spp1* are known to drive fibrosis, we aimed to quantify renal fibrosis in histological sections and observed an increased fibrotic area in the renal cortex of mice 28d post-MI compared to sham-operated controls (Fig. 3B). Next, RNA sequencing from kidney was performed on seven mice 28d post-MI, revealing a higher number of DEGs compared to day 5 post-MI, with 41 genes upregulated and 45 downregulated (Fig. 3C, Supplementary Table S6). Notably, none of the DEGs identified at 5d post-MI was significantly regulated at 28d post-MI. Subsequently, GO enrichment analysis was conducted on kidney RNA sequencing data from 28d post-MI using DEGs as input. The significantly enriched GO terms were refined using the REVIGO tool to remove redundancy. Focusing on GO terms in which more than five DEGs were annotated, 21 significantly enriched terms were identified. In Fig. 3D, the ten most significantly enriched GO terms are displayed. Among these, six – including the two terms with the lowest adjusted p-value and the highest zScore – were associated with lipid metabolism.

Since the majority of the 21 significantly enriched GO terms were linked to metabolic processes, we suggest that the observed changes may reflect an adaptive response to reduced renal perfusion and oxygen availability following MI. Furthermore, the increased fibrosis in the kidney could indicate a response to heart-mediated stimuli, potentially further contributing to renal functional impairment.

### Impact of kidney dysfunction on cardiac remodelling in the chronic post-MI phase

**Fig. 4:**
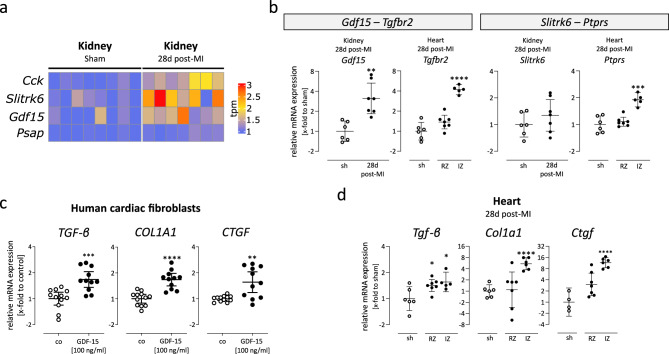
Upregulated ligands in the kidney and their effects on cardiac fibrosis. (**a**) The heatmap displays significantly upregulated ligands in the kidney. RNA sequencing data (tpm counts) is normalized to the mean of sham controls and shown separately for each sample. DEGs were identified using DESeq2 with the Wald test and Benjamini–Hochberg correction (adjusted *p*-value < 0.05). (**b**) Gene expression analysis of two inferred ligand-receptor pairs in the kidney and heart. Gene expression was normalized to *Cdkn1b* and sham controls. *Gdf15* was significantly upregulated in the kidney 28d post-MI, and its receptor *Tgfbr2* was significantly upregulated in the IZ. Significance was tested using an unpaired two-tailed *t*-test with Bonferroni correction. (**c**) Human cardiac fibroblasts, derived from endomyocardial biopsies, were stimulated with 100 ng/ml GDF-15 for 24 h. Gene expression of fibrotic markers *TGF-β*, *COL1A1*, and *CTGF* was normalized to *18S* and untreated controls. All genes were significantly upregulated following GDF-15 treatment (unpaired two-tailed t-test) (**d**) Gene expression analysis of fibrotic markers *Tgf-β*, *Col1a1*, and *Ctgf* in the LV tissue 28d post-MI. *Tgf-β* was significantly upregulated in both RZ and IZ, while *Col1a1* and *Ctgf* were significantly upregulated in the IZ. Gene expression was normalized to *Cdkn1b* and sham controls. Significance was tested using an unpaired two-tailed *t*-test with Bonferroni correction. For some genes, IZ data is missing due to low RNA yield. For all gene expression analyses, data are plotted as 2^−ΔΔCt^ (geometric mean ± 95% CI; *, **, ***, ****; *P* < 0.05, 0.01, 0.001, 0.0001).

Next, we aimed to investigate the impact of secondary affected kidney 28d post-MI on the heart in the chronic phase after MI. Therefore, we examined whether any genes encoding secreted ligands were significantly upregulated in the kidney 28d post-MI (41 upregulated DEGs, Fig. 3C). Using the ligand-receptor interaction database of *CellChat*, we identified four significantly upregulated ligands (*Cck, Slitrk6, Gdf15, Psap*) in the kidney 28d post-MI, with their gene expression visualized as heatmap in Fig. 4A. While *Cck*, *Slitrk6* and *Gdf15* showed a more than 1.4-fold increase in expression, the upregulation of *Psap* was modest (1.1-fold) compared to controls. In the publicly available human single-cell kidney dataset,^21^ *CCK* and *SLITRK6* were predominantly expressed in *pelvic epithelial cells* (Supplementary Fig. 3A). *GDF15* was detected in several cell types, including *pelvic epithelial cells*, *connecting tubule*, *transitional urothelium*, and fibroblasts. *PSAP* was also expressed in multiple cell populations, with particularly strong expression in monocytes and macrophages.

We aimed to investigate the expression of their respective receptors in the heart, which were according to the *CellChat* database CCKAR*,* PTPRS*,* TGFBR2 and GPR37. Based on the basal gene expression in our RNA sequencing data of sham hearts, we chose to investigate the interactions between *Gdf15*-*Tgfbr2* and *Slitrk6*-*Ptprs*, as the other two receptors showed only minimal expression. This is consistent with the publicly available human single-cell dataset from Kuppe et al.,^22^ in which *CCKAR* was not detected and *GPR37* showed only minimal expression in all cardiac cells. In contrast, *TGFBR2* and *PTPRS* were highly expressed across multiple cardiac cell types (Supplementary Fig. 3B).

We quantified the gene expression of these two receptors in the corresponding hearts of the mice from which the kidneys were used for RNA sequencing. The gene expression analysis of *Tgfbr2* and *Ptprs* was significantly increased in the IZ 28d post-MI compared to sham. Additionally, gene expression analysis confirmed that *Gdf15* was significantly elevated in the kidney 28d post-MI (Fig. 4B) validating the RNA sequencing data. To test this interaction in vitro, we used recombinant human GDF-15 to treat human cardiac fibroblasts derived from endomyocardial biopsies as previously described.^16^ As shown in Fig. 4C, pro-fibrotic genes were upregulated in cardiac fibroblasts after stimulation with GDF-15, which in turn might contribute to the aggravation of cardiac dysfunction following an initially cardiac-induced renal damage. In line with this, in mice, *Tgf-β* was significantly elevated in the RZ and IZ, and *Col1a1* and *Ctgf* were strongly elevated in the IZ 28d post-MI (Fig. 4D).

## Discussion

Signalling programs and circulating biomolecules mutually aggravate heart and kidney dysfunction. Here, we aimed to infer interorgan signalling between heart and kidneys after MI in mice based on RNA sequencing to identify secreted biomolecules driving CRS. Our salient findings are: (1) Only minor changes in gene expression were detected in the kidney during the acute phase 5d post-MI, however several genes inferred to target kidney receptors were highly upregulated in the IZ, with *Postn* and *Spp1* being the most probable ligands. (2) During the chronic phase 28d post-MI, the kidney showed increased fibrosis compared to sham kidneys and a higher number of DEGs than at 5d post-MI, these DEGs indicate an altered lipid metabolism. (3) Based on upregulated DEGs in kidney at 28d post-MI, we inferred two kidney-to-heart interactions: *Slitrk6*-*Ptprs* and *Gdf15*-*Tgfbr2.* We experimentally investigated the latter in vitro, demonstrating that GDF-15 stimulation is leading to increased pro-fibrotic gene expression in human cardiac fibroblasts.

First, we investigated alterations in gene expression 5d post-MI in heart and kidney using RNA sequencing. While more than 11,000 DEGs were detected in the IZ of the heart, in kidney interestingly, only 18 genes were significantly dysregulated, suggesting that DAMPs released early after MI have a limited impact on the kidney at 5d post-MI. Among the ten upregulated genes, five were hemoglobin-related, indicating a potential response to hypoxia or erythrocyte infiltration. This was further supported by significantly enriched GO terms such as “hydrogen peroxide catabolic process.” Additionally, *Ccn1* was downregulated 5d post-MI. As a well-known stress-responsive matricellular protein, *Ccn1* has been implicated in oxidative stress modulation, tissue remodelling, and apoptosis.^23^ Its expression is known to increase under hypoxic conditions and has been shown to induce reactive oxygen species (ROS) production.^24–26^ However, in our dataset, *Ccn1* expression was significantly reduced in kidney suggesting that post-MI stress responses in the kidney may differ from those observed in other models. The downregulation of *Ccn1* could indicate an alternative adaptive mechanism, possibly reducing oxidative stress or modulating extracellular matrix remodelling in a different manner. While CCN1 has been reported to be upregulated in kidney early after renal ischemia–reperfusion injury,^27^ in our dataset its downregulation after ischemic injury of the heart suggests that the kidney’s response to post-MI stress may follow a distinct regulatory pattern.

Based on our RNA sequencing data from heart and kidney, we aimed to infer potential interorgan interactions using *CellChat*. Since the highest number of interactions were inferred from or to the IZ, we concluded that the inferred interactions were primarily driven by upregulated ligands and receptors in the injured heart, while both organs act as senders as well as receivers. As a result of the infarction, more than 5000 genes were upregulated in cardiac tissue, with *Postn* and *Spp1* (or the coding protein OPN) emerging as key mediators of heart-to-kidney signalling. Both genes are known to be strongly upregulated following MI,^28–30^ which is in line with our data, in which *Postn* and *Spp1* were significantly upregulated in both the IZ and RZ 5d post-MI. In the kidney, *SPP1*/OPN is participating in various pathological processes and has been linked to increased fibrosis in certain forms of renal injury.^31^ Notably, baseline OPN plasma levels in patients with acute kidney injury were significantly higher compared to controls.^32^ Moreover, elevated OPN plasma levels correlate with multivessel coronary artery disease, progressive renal dysfunction, and an increased risk of adverse cardiovascular events in patients with CKD.^32,33^ Steinbrenner et al. demonstrated in the large German Chronic Kidney Disease cohort that higher OPN plasma concentrations are strongly associated with lower eGFR and higher albuminuria, and, similarly, in a cohort of CKD patients with acute HF, OPN was shown to be independently associated with worsening renal function.^34,35^ Similarly, POSTN has been identified as a player in renal tissue remodelling, contributing to both inflammation and fibrosis.^36^ Blocking POSTN prevented inflammation in an acute kidney injury mouse model, while its overexpression in the collecting duct promoted cyst growth and fibrosis, accelerating renal function decline.^37,38^ Conversely, *Postn* knockout in mice reduced fibrosis and preserved renal function, highlighting its potential as a therapeutic target in CKD.^39^.

Given that both POSTN and *SPP1*/OPN are potent drivers of fibrosis, we investigated renal fibrosis 28d post-MI and found a significant increase. Previous studies have demonstrated that MI can induce renal fibrosis, with a concurrent decline in glomerular filtration rate as early as three days post-MI.^40^ This functional impairment was further reflected by elevated serum creatinine and blood urea nitrogen levels, indicating progressive renal dysfunction.^40^ Nevertheless, POSTN and *SPP1*/OPN are pleiotropic molecules with known systemic actions affecting multiple organs and therefore inferred signalling axis is probably not restricted to the heart-kidney interaction.

The subsequent RNA sequencing of kidneys 28d post-MI revealed more DEGs compared to 5d post-MI, indicating a long-term influence on kidney after MI. GO term analysis highlighted profound alterations in renal lipid metabolism at 28 days, particularly within the terms “lipid catabolic process” and “lipid oxidation,” consistent with a metabolic adaptation to persistent ischemia and energy stress. The most downregulated gene was *Lipe*, encoding hormone-sensitive lipase, a key enzyme in intracellular lipolysis. Similarly, *Pnpla2*, encoding adipose triglyceride lipase (ATGL), was downregulated, and all other annotated DEGs in the term “lipid catabolic process” showed reduced expression. This pattern suggests a suppression of intracellular lipolysis, resulting in decreased availability of free fatty acids for beta-oxidation in mitochondria. Such a mechanism aligns with lipid accumulation observed in renal cells in both experimental and human studies.^41,42^ Consistently, genes linked to the GO term “lipid oxidation” were also predominantly downregulated, indicating reduced fatty-acid β-oxidation, a metabolic signature that has been described in mouse and human fibrotic kidneys.^41^ There are only a few studies suggesting that *Spp1*/OPN and POSTN signalling may be involved in lipid metabolic processes in the kidney.^43–45^ In mouse models of genetic kidney disease, OPN-deficiency markedly reduced lipid accumulation in renal tubules compared with diseased wildtype controls.^44^ Similarly, APOE^−/−^OPN^−/−^ double-knockout mice showed less renal lipid deposition than wildtype mice, indicating that OPN might promote lipid retention under pathological conditions.^45^ In addition, *Postn*-deficient aged mice displayed decreased renal lipid accumulation compared to age-matched wildtype controls.^43^.

It is well established that renal dysfunction is associated with changes in lipid metabolism, which, in turn, may contribute to cardiovascular complications, suggesting a potential feedback loop between heart and kidney dysfunction.^46–49^ However, while metabolic alterations in the kidney following MI or in the context of HF are known to occur, their exact mechanisms and implications remain poorly understood. At least in part, they might be induced by systemic activation of the renin–angiotensin–aldosterone system (RAAS), which occurs in response to reduced cardiac output and renal perfusion.^50^ Elevated levels of angiotensin II and aldosterone promote vasoconstriction, inflammation, and fibroblast activation, thereby contributing to interstitial fibrosis in the kidney after MI.^50^ Such mechanisms may amplify the adverse feedback loop between cardiac injury and renal dysfunction. These observations may also have therapeutic implications, as pharmacological inhibition of the RAAS or mineralocorticoid receptor blockade has been shown to attenuate cardiac and renal fibrosis in experimental and clinical settings.^50,51^ Such interventions might therefore represent potential strategies to mitigate the maladaptive cardio–renal signalling observed after MI.

Overall, the renal response following MI is likely influenced by a combination of hemodynamic and humoral factors. While reduced cardiac output and renal perfusion can induce hypoxic and fibrotic signalling, circulating stress mediators released from the injured myocardium may further modulate renal transcriptional programs. As our analysis is based solely on transcriptomic data, these mechanisms cannot be disentangled, and additional physiological and proteomic measurements will be necessary to establish causal links.

After 28d post-MI, we aimed to generate hypotheses on interorgan signalling from the kidney to the heart and identified four upregulated genes that were classified as ligands in the *CellChat* database. Of the four upregulated inferred ligands, GDF-15 is known to play a significant role in heart failure, particularly in the context of CKD were it was shown that GDF-15 is associated with adverse outcome.^52^ In the CANVAS trial, each doubling of baseline GDF-15 plasma levels was linked to a higher risk of cardiovascular events, HF, and kidney complications.^53^ In our study, *Gdf15* is significantly upregulated in the kidney 28d post-MI. In addition, we found that the receptor *Tgfbr2* is expressed in the heart 28d post-MI, with significant upregulation in the IZ. In line with this, we confirmed in vitro that GDF-15 treatment increased the expression of fibrosis-related genes in human cardiac fibroblasts. Supporting this observation, several studies have demonstrated pro-fibrotic actions of GDF-15. Here, GDF-15 stimulation induced pro-fibrotic gene expression and myoblast transformation in two different fibroblast cell lines.^54,55^ Additionally, overexpression of GDF-15 in rat cardiac fibroblasts increased expression of fibrosis markers.^56^ Several studies have highlighted the association between GDF-15 and cardiac fibrosis in the context of both HF and MI.^57–59^ In a recently published large cohort study of adults presenting to the emergency department with suspected MI, circulating GDF-15 levels were independently associated with subsequent 30-day and 90-day all-cause mortality or recurrent acute MI.^60^ Lok and colleagues measured circulating GDF-15 levels before and after LVAD implantation and prior to heart transplantation or explantation. The results indicated that circulating GDF-15 was significantly correlated with kidney function and the severity of myocardial fibrosis, although gene and protein expression of GDF-15 were barely detectable in the myocardium.^58^ This suggests that GDF-15 originates from another source. Furthermore, Wang et al. demonstrated that both focal and diffuse fibrosis were associated with GDF-15 levels in HF patients.^57^ Given the pleiotropic nature and broad involvement in diverse pathophysiological pathways – including inflammation, metabolic stress, and tissue injury – we do not consider GDF-15 to represent a unique or organ-specific signalling axis between the heart and kidney. Rather, its upregulation likely reflects a systemic stress response that may contribute to, but is not exclusive to, the cardiorenal context. Moreover, our study is limited to transcriptomic analyses and proposes candidate molecules, such as GDF-15, whose circulating levels and functional relevance should be investigated in future studies on cardiorenal syndrome.

## Conclusion

The interplay between heart and kidney following MI reveals a complex pathophysiological relationship that significantly impacts both cardiac and renal function. As myocardial injury accelerates kidney dysfunction, heart-kidney crosstalk contributes to the development of CRS, further complicating patient outcomes. Conversely, renal dysfunction can exacerbate cardiovascular diseases, highlighting the bidirectional nature of the relationship between these two organs. Investigating the molecular mechanisms of this crosstalk may open new avenues for improving treatment strategies and patient prognosis.

Here, we conducted an exploratory, bioinformatic-based analysis of heart-kidney interorgan crosstalk after MI in mice. The data suggest that MI does not have major effects on the kidney in the acute setting, but rather induces long-term changes associated with chronic HF, indicative of CRS type 2 mimicking the clinical course of post-MI patients. Our findings propose factors that may mediate the development of CRS after MI. These are promising candidates for further in-depth mechanistic and clinical investigations of CRS exacerbation, especially after a primary cardiac event. We acknowledge that transcriptome-based inference of ligand–receptor communication provides an integrative but indirect view of interorgan signalling. While this approach highlights potential molecular candidates, confirmation at the protein and functional level in future studies will be essential to establish causality.

## Limitations

Permanent LAD ligation represents a non-reperfused model of human myocardial infarction. However, this approach enables the formation of a large, clearly demarcated infarct scar in every animal, ensuring reproducible sampling of macroscopically homogeneous tissue across biological replicates. This consistency allowed us to focus on robust transcriptional remodelling within the infarcted myocardium, while recognizing that the model does not capture the reperfusion and regional heterogeneity typical of clinical MI. While our transcriptomic data of the kidney might capture the sustained remodelling process associated with chronic HF, future studies in reperfused models will be important to validate our data under more clinically relevant MI conditions.

Because cardiac RNA sequencing data at 28d post-MI were unavailable, we could not perform a full bidirectional *CellChat* analysis at this later stage. Moreover, our proposed ligand–receptor interactions are based solely on transcriptomic inference and therefore provide an indirect perspective on interorgan communication. Experimental validation at the protein and functional level will be required to substantiate these proposed molecular interactions.

## Supplementary Information

Supplementary Information 1.

Supplementary Information 2.

Supplementary Information 3.

Supplementary Information 4.

Supplementary Information 5.

Supplementary Information 6.

Supplementary Information 7.

## Data Availability

The datasets generated and analysed during the current study are available at GEO repository under the accession numbers (GSE307265 and GSE307268).
