# Supplementary material for: Systems biology approach uncovers candidates for kidney-heart interorgan crosstalk after myocardial infarction
Source: Sci Rep. 2025 Dec 5;15:43267. doi: 10.1038/s41598-025-30712-z (PMC12686400; doi:10.1038/s41598-025-30712-z)
Supplement: Supplementary file 7 — Supplementary Information 7. [file 41598_2025_30712_MOESM7_ESM.pdf]

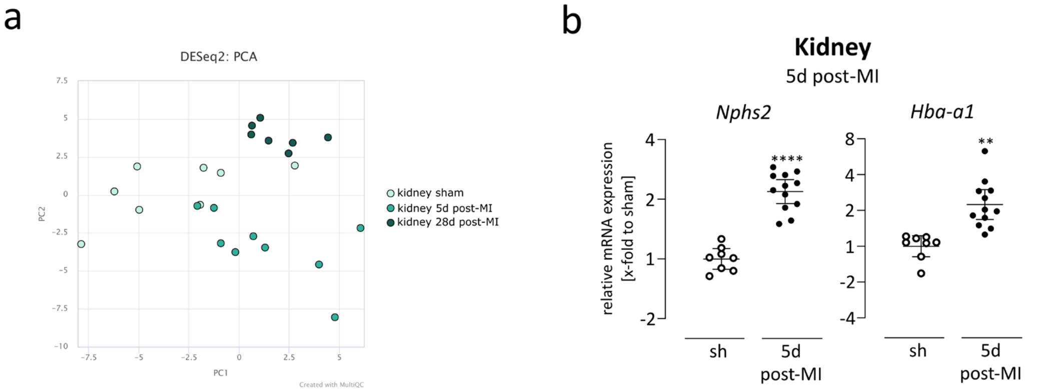

Supplementary Figure 1: Validation of RNA sequencing results in the kidney 5d post-MI

a: Principal component analysis (PCA) of variance-stabilized DESeq2 counts, visualized through MultiQC (v1.16).
b: Gene expression of *Nphs2* and *Hba-a1* was measured in the kidney 5d post-MI. Gene expression was normalized to Cdkn1b and sham controls. Gene expression data are plotted as $2^{-\Delta\Delta Ct}$ (geometric mean ± 95% CI, unpaired two-tailed t-test with Bonferroni correction.; **, ****; P < 0.01, 0.0001).

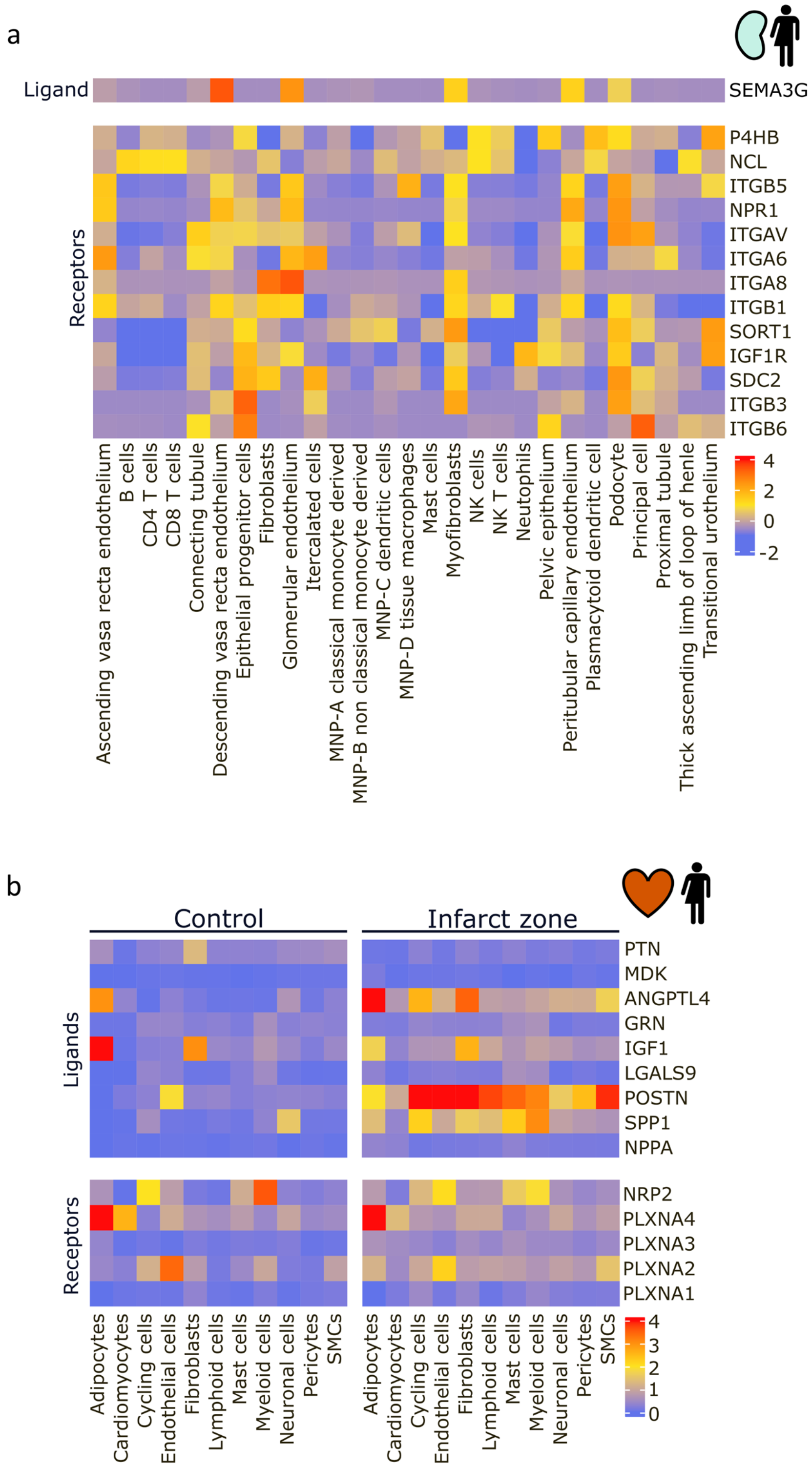

Supplementary Figure 2: Cell type–specific expression in human kidney and heart single-cell datasets of inferred ligands and receptors 5d post-MI in mice.

a: Expression of ligand and receptors inferred from 5d post-MI mouse data (Fig. 2) in publicly available human kidney single-cell RNA-sequencing dataset published by Stewart et al[1]

b: Expression of ligand and receptors inferred from 5d post-MI mouse data (Fig. 2) in publicly available human cardiac single-cell RNA-sequencing dataset including control and myocardial infarction samples published by Kuppe et al[2]

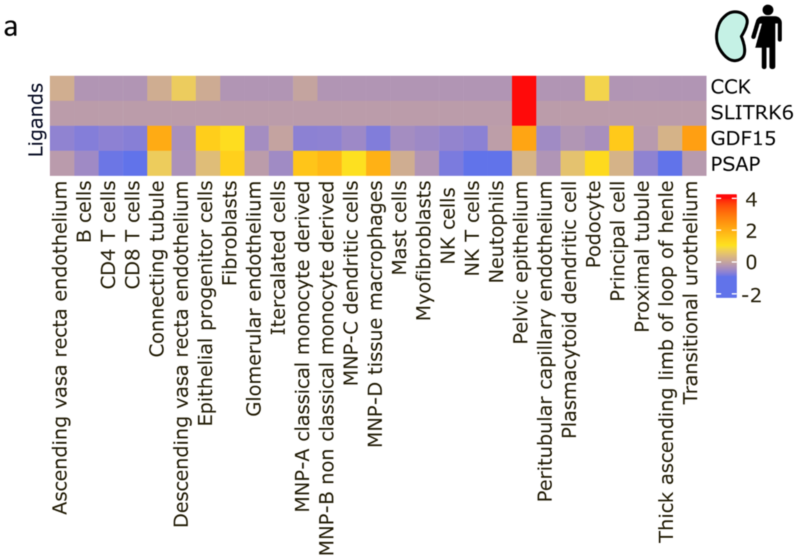

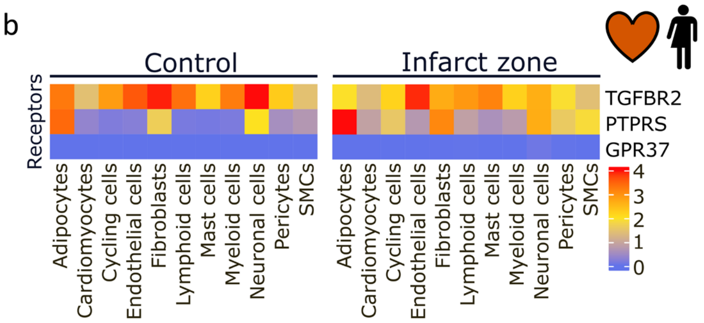

Supplementary Figure 3: Cell type–specific expression in human kidney and heart single-cell datasets of inferred ligands and receptors 28d post-MI in mice.

a: Expression of ligands inferred from 28d post-MI mouse data (Fig. 4) in publicly available human kidney single-cell RNA-sequencing dataset published by Stewart et al[1]

b: Expression of receptors inferred from the 28d post-MI mouse data (Fig. 4) in publicly available human cardiac single-cell RNA-sequencing dataset including control and myocardial infarction samples published by Kuppe et al[2]

References

1 Stewart, B. J. *et al.* Spatiotemporal immune zonation of the human kidney. *Science* **365**, 1461-1466, doi:10.1126/science.aat5031 (2019).

2 Kuppe, C. *et al.* Spatial multi-omic map of human myocardial infarction. *Nature* **608**, 766-777, doi:10.1038/s41586-022-05060-x (2022).
